# Plant BTB (Broad-Complex, Tramtrack, and Bric-à-Brac) Proteins: Structural Features, Biological Functions, and Roles in Stress Responses

**DOI:** 10.3390/plants15071059

**Published:** 2026-03-30

**Authors:** Ying Zhang, Jiadong Xie, Kaixuan Dai, Yanchun Yu, Limin Wu

**Affiliations:** College of Life and Environmental Sciences, Hangzhou Normal University, Hangzhou 311121, China; 2023111010047@stu.hznu.edu.cn (Y.Z.); 2023112010047@stu.hznu.edu.cn (J.X.); 2024111010051@stu.hznu.edu.cn (K.D.)

**Keywords:** BTB protein, drought stress, heat stress, cold stress, salt stress, light, plant development

## Abstract

As sessile organisms, plants must continuously perceive and integrate external environmental cues with internal developmental signals to optimize growth, reproduction, and survival. Central to this adaptive capacity is the ubiquitin-proteasome system (UPS), the primary pathway for selective protein degradation in eukaryotes. Within the UPS, BTB (Broad-Complex, Tramtrack, and Bric-à-brac) proteins serve as critical substrate adaptors for the Cullin3 (CUL3)-based E3 ubiquitin ligase complex. These proteins play indispensable roles in plant growth, development, hormone signaling, and responses to abiotic stresses. Recent advances have revealed the remarkable functional versatility of BTB proteins, implicating them in the regulation of photomorphogenesis, root architecture, flowering time, stress resilience, and yield-related traits. With 80 BTB-encoding genes in *Arabidopsis thaliana* and key orthologs identified in major crops—including of rice (*Oryza sativa*), soybean (*Glycine max*), and maize (*Zea mays*)—BTB proteins act as molecular “bridges” that integrate developmental programs with environmental stress signals. This review summarizes the structural features, classification, and multifaceted functions of plant BTB proteins, with an emphasis on their roles in growth regulation, abiotic stress tolerance, light signaling, and agricultural productivity. We further discuss their mechanisms in ubiquitin-dependent proteolysis, transcriptional regulation, and signal integration, offering insights into their potential as targets for engineering climate-resilient crops and advancing sustainable agriculture.

## 1. Introduction

Global climate change has intensified the frequency and severity of abiotic stresses—such as drought, salinity, and extreme temperatures—posing unprecedented challenges to sustainable agriculture [1]. As sessile organisms, plants have evolved sophisticated molecular regulatory networks to sense, integrate, and respond to dynamic environmental fluctuations alongside endogenous developmental cues [2,3,4]. Among these regulatory systems, the ubiquitin-proteasome system (UPS) serves as a central hub for post-translational control, orchestrating the selective degradation of key regulatory proteins to modulate growth and development, hormone signaling, and stress adaptation [5,6].

A pivotal component of the UPS in plants is the Cullin 3 (CUL3)-based E3 ubiquitin ligase complexes, in which BTB (Broad-Complex, Tramtrack, and Bric-à-brac) proteins function as substrate recognition subunits [7,8,9]. Characterized by a conserved ~120-amino-acid BTB/POZ domain, these proteins often harbor additional C- or N-terminal domains—such as MATH, ANK, or TAZ—that confer functional specificity through diverse protein–protein interactions [10,11]. Consequently, BTB proteins occupy central nodes in regulatory networks governing virtually every aspect of plant life.

Recent functional studies have elucidated the pleiotropic roles of BTB proteins throughout the plant life cycle [12,13,14,15]. They precisely regulate photomorphogenesis, root system development, flowering time, and seed maturation by targeting key regulators—such as PIF3—for ubiquitin-mediated degradation [16,17,18,19,20,21]. In stress adaptation, BTB proteins enhance tolerance to drought, salinity, and temperature extremes by modulating abscisic acid (ABA) signaling, reactive oxygen species (ROS) scavenging, and ion homeostasis [22,23,24]. Like other large transcription factor families (e.g., MYB, WRKY), the BTB protein family has undergone extensive expansion and functional diversification across plant lineages [25]. This reflects lineage-specific adaptations [26]. Notably, crop BTB proteins have been linked to agronomic traits such as stress tolerance and yield, with some already explored in breeding programs [27,28,29].

Collectively, these findings position BTB proteins as dynamic integrators—“molecular bridges” that link developmental programs with environmental stress responses. This review provides a systematic overview of the structural architecture, evolutionary classification, and functional diversity of plant BTB proteins. We focus on their mechanistic roles in abiotic stress responses (drought, salinity, heat and cold) and key developmental processes, such as photomorphogenesis, root development, and flowering regulation. Central to this discussion is their role as substrate recognition subunits of the Cullin-RING Ligase 3 (CRL3) complex, illustrating how they integrate environmental signals with hormonal networks via targeted ubiquitination. By synthesizing recent breakthroughs in both model plants and crops, we explore the application potential of BTB proteins as “molecular switches” in future crop breeding for stress resistance and quality improvement. Finally, we outline critical unresolved questions—such as substrate recognition specificity, functional redundancy, and cross-species conservation—to provide a theoretical foundation for breeding climate-smart crops and ensuring global food security.

## 2. BTBs Proteins in Plants

**Figure 1:**
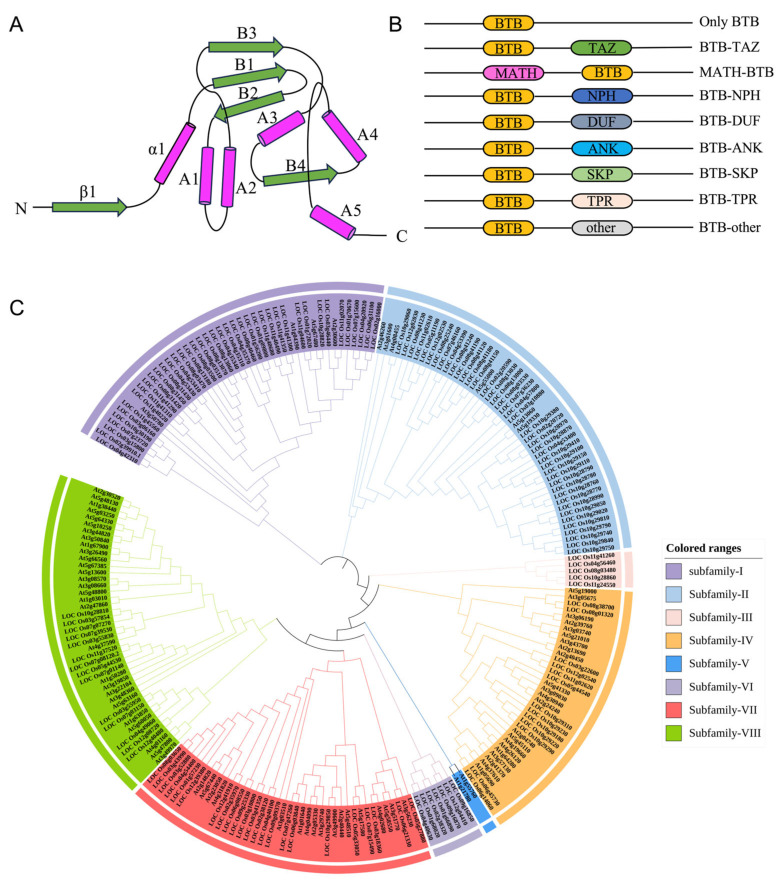
Structural characteristics, domain architectures, and phylogenetic analysis of plant BTB proteins. (**A**) Schematic diagram of the topology of the BTB/POZ domain. The domain typically assumes a characteristic fold consisting of a cluster of α-helices (A1–A5), which drive dimerization, and central β-sheets (B1–B4) that enhance structural stability. Green arrows and pink cylinders indicate β-sheets and α-helices, respectively. (**B**) Domain architectures of the plant BTB protein family. BTB proteins are classified into distinct subfamilies based on the diverse auxiliary domains (e.g., TAZ, MATH, NPH, ANK, SKP, TPR, and DUF) coupled to the N- or C-terminus of the core BTB domain. Different colors represent distinct auxiliary domains. (**C**) Phylogenetic tree of BTB proteins from *Arabidopsis thaliana* and *Oryza sativa*. The tree was constructed using MEGA11 software based on protein sequences retrieved from the NCBI database. The proteins are clustered into eight major subfamilies (Subfamily I–VIII) according to variations in the N-terminal BTB domain and C-terminal conserved domains, as indicated by the different colored ranges.

The BTB (Broad-Complex, Tramtrack, and Bric-à-brac) protein family is ubiquitously distributed across eukaryotes and is defined by the presence of a conserved BTB/POZ domain [30]. In plants, BTB proteins act as pivotal regulators of growth, development, immune defense, and stress responses through their involvement in the ubiquitin-proteasome system (UPS), transcriptional regulation, and signal transduction [31]. The BTB/POZ domain comprises approximately 120 amino acids and adopts a characteristic α/β-fold topology. Structurally, it consists of a cluster of five α-helices (A1–A5) that mediate homodimerization or heterodimerization, a central region of antiparallel β-sheets (B1–B4) that confers structural stability, and an N-terminal extension that interacts with Cullin 3 (CUL3) (Figure 1A). Together, these elements form a unique “bowtie”-shaped conformation that provides the structural basis for substrate recognition and multiprotein complex assembly [10,32,33]. BTB domains typically exist as dimers and serve as scaffolds for higher-order complexes [34].

Beyond the core domain, plant BTB proteins are flanked by diverse auxiliary domains at their N- or C-termini, giving rise to functionally distinct subfamilies, including BTB-TAZ, MATH-BTB, BTB-NPH, BTB-ANK, BTB-Skp, BTB-DUF, and BTB-TPR [12] (Figure 1B). These auxiliary domains confer distinct functional specificities to the BTB proteins, as summarized in Table 1.

The plant BTB protein family exhibits not only extensive structural diversity but also striking variation in gene family size across species. Phylogenetic analysis reveals that the *Arabidopsis thaliana* genome encodes approximately 80 BTB proteins, whereas the monocot *Oryza sativa* (rice) harbors as many as 149 members [22]. Based on sequence divergence in the N-terminal BTB domain and the identity of C-terminal auxiliary domains, BTB proteins from both species can be broadly classified into eight distinct subfamilies (Figure 1C)—a structural diversity that directly dictates their functional specificity [22]. This pronounced difference in gene number suggests that the BTB gene family underwent extensive gene expansion events during monocot evolution, likely driven by selective pressures associated with developmental complexity and environmental adaptation. Phylogenetic tree reconstruction further reveals that certain BTB proteins from *Arabidopsis* and rice cluster within shared evolutionary clades, forming orthologous groups that likely originated from a common ancestor and have retained conserved functions. At the same time, rice displays prominent lineage-specific expansion branches, indicating that numerous BTB genes acquired novel or specialized roles following speciation to meet unique physiological and ecological demands.

This evolutionary and structural diversification is reflected in their biological functions. In *Arabidopsis*, for example, AtBPM proteins (containing MATH and BTB domains) act as substrate adaptors in CRL3 E3 ubiquitin ligase complexes to mediate the degradation of transcription factors involved in photomorphogenesis and hormone signaling [46,47]. By contrast, other BTB proteins function directly as transcriptional regulators, modulating processes such as flowering time and leaf morphogenesis [46,47]. Similarly, rice BTB proteins display high functional versatility, contributing to the regulation of key agronomic traits, including abiotic stress tolerance, drought adaptation, and panicle development [48].

In summary, the plant BTB family has undergone substantial expansion and structural innovation throughout its evolutionary trajectory. The combinatorial diversity of N-terminal BTB domains and C-terminal auxiliary motifs serves as the primary molecular basis for functional differentiation. Whether acting as substrate recognition modules in ubiquitin-mediated proteolysis or directly as transcriptional regulators, BTB proteins are indispensable across nearly all aspects of plant life—from vegetative growth and reproductive development to environmental stress resilience.

## 3. Functions of BTB Proteins

### 3.1. Roles in Abiotic Stress Responses

Building upon the extensive structural diversification and evolutionary expansion detailed above, BTB proteins function as the specialized substrate-recruiting modules that confer functional specificity to Cullin-RING Ligase 3 (CRL3) complexes. While Section 2 highlighted their structural architecture, it is their dynamic role within the ubiquitin-proteasome system (UPS) that translates these structural features into precise physiological outputs. Rather than serving merely as static structural bridges between Cullin 3 and substrates, BTB proteins act as dynamic “molecular governors” that orchestrate targeted proteolysis to maintain cellular homeostasis [49,50].

Recent mechanistic advances have revealed that BTB-mediated regulation operates primarily through two sophisticated modes to navigate the intricate balance between plant growth and environmental adaptation [51,52]. First, functioning as rapid signal transducers, BTB proteins modulate the stability of master transcription factors and signaling kinases to swiftly switch gene regulatory networks. Second, they participate in metabolic reconfiguration by targeting key biosynthetic enzymes for degradation, directly altering metabolic fluxes to support survival. This multilayered regulatory capacity positions the BTB family as a central node in the “growth-defense trade-off”, allowing plants to minimize yield penalties while maximizing stress resilience [53].

The following sections will systematically dissect the specific roles of BTB proteins in responding to abiotic challenges (drought, heat, cold, salt) and regulating key developmental transitions.

#### 3.1.1. Role of BTB Proteins in Plant Drought Stress

Drought is a prevalent abiotic stress that induces severe cellular dehydration, reactive oxygen species (ROS) accumulation, and metabolic dysfunction [54]. Furthermore, this stress significantly impairs vegetative growth [55,56,57]. Ultimately, these physiological disruptions cause substantial crop yield losses [58]. To counteract these challenges, plants deploy multifaceted adaptive strategies, including stomatal closure, osmoprotectant accumulation, and the activation of antioxidant and abscisic acid (ABA) signaling systems [59,60,61]. Among the key regulatory components orchestrating these physiological and molecular responses, BTB proteins function as pivotal molecular hubs that directly integrate multiple drought-resistance pathways.

Abscisic acid (ABA) serves as a central phytohormone in drought responses, governing stomatal closure, growth suppression, and the transcriptional activation of drought-responsive genes [62,63]. Accumulating evidence indicates that BTB proteins are integral modulators of ABA signaling. In *Arabidopsis thaliana*, the AtBTB-A2s proteins act as negative regulators of this pathway by interacting with SnRK2.6 (also known as OST1), a core kinase in ABA signal transduction. This interaction inhibits SnRK2.6 kinase activity, thereby attenuating the expression of downstream drought-responsive genes (e.g., RD29A/B, RAB18, ABI5) and impairing ABA-induced stomatal closure. Consequently, plants exhibit reduced drought tolerance [22]. Elucidating the precise molecular mechanisms by which AtBTB-A2s precisely modulates SnRK2.6 activity under drought conditions remains a critical avenue for future investigation.

BTB proteins actively modulate reactive oxygen species (ROS) homeostasis and secondary metabolism to mitigate drought-induced cellular injury [64]. For example, in apple (*Malus domestica*), the BTB protein MdBT2 serves as a master repressor that coordinately controls anthocyanin biosynthesis and ROS scavenging [65]. Under non-stress conditions, MdBT2 acts as a substrate adapter for CRL3 complexes to target multiple positive regulators (e.g., MdMYB1, MdMYB9, MdbZIP44, MdERF38, and MdbHLH160) for ubiquitin-mediated degradation. This continuous clearance effectively prevents the unnecessary allocation of metabolic resources to defense responses in the absence of stress [66,67].

Under drought stress, however, ABA signaling is activated and strongly represses MdBT2 expression. The resulting decline in MdBT2 protein levels alleviates repression of its downstream targets, enabling the stabilization and accumulation of transcription factors such as MdbHLH160, MdMYB1, MdMYB9, MdHDZ27, MdbZIP44 and MdERF38. These factors subsequently activate the expression of anthocyanin biosynthetic genes, antioxidant enzyme genes (e.g., MdSOD1 and MdDREB2A-like), and canonical drought-tolerance genes (MdRD29A and MdRD29B). This cascade enhances anthocyanin accumulation and boosts SOD activity, facilitating efficient scavenging of drought-induced ROS and ultimately improving drought tolerance in apple. Through this elegant “derepression” mechanism, MdBT2 functionally couples ABA signaling with the antioxidant defense system [68,69]. Moreover, MdBT2 further fine-tunes drought responses by mediating the ubiquitination and degradation of the transcription factor MdNAC143, a positive regulator of drought-adaptive pathways. By suppressing MdNAC143 activity, MdBT2 adds another layer of negative regulation to the plant’s drought response network [69,70,71,72].

Importantly, the role of BTB proteins in drought tolerance extends beyond ABA and ROS signaling. In sweet potato (*Ipomoea batatas*), overexpression of the IbBT4 activates multiple genes involved in brassinosteroid (BR) signaling, proline biosynthesis (e.g., P5CS and P5CR), and ROS detoxification (e.g., SOD, GPX, APX, and CAT). Transgenic *Arabidopsis* lines expressing IbBT4 exhibit markedly enhanced drought tolerance, underscoring the capacity of BTB proteins to serve as integrative nodes that coordinate crosstalk among distinct hormonal and metabolic stress-response pathways [73,74]. Similarly, transcriptomic analyses in soybean (*Glycine max*) have revealed significant upregulation of multiple GmBTB genes under drought stress, suggesting their functional relevance in crop drought resilience [27].

**Figure 2:**
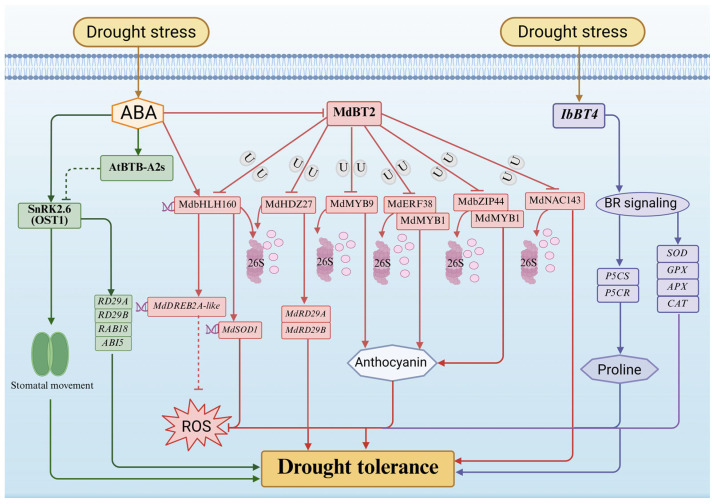
Proposed working model of BTB proteins regulating drought tolerance in plants. AtBTB-A2s negatively regulate tolerance by inhibiting the kinase SnRK2.6, thereby blocking stomatal closure and drought-responsive gene expression (RD29A/B, RAB18, ABI5). Drought-induced ABA represses MdBT2 expression. This stabilizes downstream transcription factors (e.g., MdbHLH160, MdMYB1, MdMYB9, MdHDZ27, MdbZIP44, MdERF38, MdNAC143), which subsequently activate antioxidant (MdSOD1, MdDREB2A-like) and dehydration-responsive (MdRD29A/B) genes to scavenge ROS. IbBT4 enhances tolerance by activating BR signaling, promoting proline biosynthesis (via P5CS/P5CR) and antioxidant enzyme activities. Solid lines indicate positive regulation or metabolic flows; T-bars indicate negative regulation or inhibition; dotted lines indicate indirect or potential regulation. This schematic represents a proposed working model synthesized from established literature. This figure was drawn using Microsoft PowerPoint.

While modulating stomatal dynamics and drought-responsive gene expression provides a critical defense against water deficit (Figure 2), extreme temperature fluctuations present another formidable challenge wherein BTB proteins similarly orchestrate complex protective mechanisms.

#### 3.1.2. Role of BTB Proteins in Plant Heat Stress

Driven by global climate change, heat stress severely threatens plant growth and crop yields by inducing protein misfolding and ROS bursts [75,76,77,78]. In addition, elevated temperatures severely impair photosynthetic capacity by inhibiting Rubisco and reducing carbon assimilation [79]. Furthermore, heat destabilizes the thylakoid membrane system to compromise Photosystem II function [80]. Consequently, this environmental constraint causes reproductive failure [81]. To survive elevated temperatures, plants activate multi-layered defense networks centered on maintaining protein homeostasis (proteostasis), primarily through the induction of Heat Shock Proteins (HSPs) and stress-responsive transcriptional cascades [82,83].

Within this framework, BTB proteins, particularly members of the MATH-BTB subfamily (also known as BPM proteins), serve as dynamic and pivotal regulators of thermotolerance. A central mechanism underlying their function as substrate adapters for CRL3 E3 ubiquitin ligases (CRL3) complexes, through which they precisely govern the stability of key transcription factors involved in heat responses. The most well-characterized example is the regulation of DREB2A, a master transcriptional activator of heat shock genes and a positive regulator of thermotolerance. While DREB2A is essential for activating downstream heat-responsive genes, its constitutive overexpression severely compromises plant growth. To balance defense and development, plants employ a sophisticated feedback loop: under non-stress conditions, BPM proteins continuously target DREB2A for ubiquitin-mediated degradation, maintaining its protein abundance at a low level. Upon heat stress, DREB2A transcription is rapidly induced, yet BPM-mediated degradation persists concurrently. This dynamic equilibrium ensures timely activation of thermoprotective genes while preventing the fitness costs associated with an excessive or prolonged DREB2A activity [20,84,85].

Intriguingly, BPM proteins also play intricate roles in acquired thermotolerance and thermomemory—the phenomenon whereby a prior mild heat exposure (“priming”) enhances tolerance to subsequent, more severe stress. Transcriptomic analyses showed that while the classic heat-responsive genes (DREB2A, HSFA3, HSP70, and HSP90) are strongly induced after acute heat treatment, the expression of HSFA2—a key regulator of thermomemory—and the activation of the retrotransposon ONSEN require prior heat priming. Notably, *Arabidopsis* lines overexpressing BPM1 exhibit suppressed induction of HSFA2 and ONSEN under standard stress. Paradoxically, after heat priming, these same lines exhibit enhanced thermotolerance, accompanied by elevated levels of HSP90 and DREB2A proteins and reduced ROS accumulation. These observations reveal that BPM proteins are not merely negative regulators but function as plastic, context-dependent nodes whose roles shift between the initial stress response and the establishment of acquired tolerance [85].

This regulatory logic further extends to the integration of temperature cues with developmental timing. For instance, under elevated temperatures, the BTB domain-containing protein LFH1 (Late Flowering at High temperature 1) interacts with the floral repressor SVP (SHORT VEGETATIVE PHASE). LFH1 assembles with CUL3A and the E2 conjugating enzyme UBC15 to form the CRL3^LFH1 E3 ligase complex, which promotes the ubiquitination and degradation of SVP. This relieves repression of flowering, thereby accelerating the reproductive transition—a strategic adaptation to avoid heat-induced reproductive failure [68,69,70,71,72,73,74,75,76,77,78,79,80,81,82,83,84,85,86].

Beyond their canonical role in ubiquitin-mediated proteolysis, certain BTB proteins have evolved non-canonical functions that operate independently of protein degradation. A striking example is NPR1 (Nonexpressor of Pathogenesis-Related genes 1) in *Arabidopsis*. Although best known as a master regulator of the salicylic acid (SA)-mediated immunity, NPR1 also functions as a redox-regulated molecular chaperone under heat stress. In its oligomeric form, NPR1 directly binds to heat-denatured proteins, preventing their irreversible aggregation and thereby preserving proteostasis. This chaperone activity—modulated by the cellular redox environment—highlights the remarkable functional versatility of the BTB protein family [87,88].

In summary, BTB proteins occupy a central position in plant thermotolerance through two complementary mechanisms. 

First, the canonical CRL3-mediated pathway, which dynamically controls the abundance of key regulators like DREB2A and SVP, acting as a “molecular governor” to fine-tune the amplitude and duration of heat responses;

Second, the non-canonical molecular chaperone function, exemplified by NPR1, which directly stabilizes denatured proteins and safeguards cellular integrity.

These dual roles underscore the importance of BTB proteins not only in acute stress mitigation but also in long-term adaptive strategies such as thermomemory and developmental plasticity. As such, they present high-value targets for molecular breeding aimed at developing climate-resilient crops. Future research should focus on dissecting the intricate regulatory networks governing BTB-mediated thermomemory and their crosstalk with other abiotic and biotic stress pathways—efforts that will be critical for ensuring global food security in an era of rising temperatures.

#### 3.1.3. Role of BTB Proteins in Plant Cold Stress

Low-temperature stress broadly encompasses both chilling and freezing conditions [89]. This environmental constraint restricts plant geographical distribution by causing membrane rigidification and oxidative damage [90]. Ultimately, these cellular injuries lead to significant reproductive impairment and yield losses [91,92,93,94].

To adapt, plants have evolved sophisticated cold acclimation mechanisms, predominantly centered on the C-repeat Binding Factor (CBF) signaling pathway, which activates cold-regulated (COR) genes to enhance freezing tolerance [95]. Serving as critical adapter factors for CRL3 E3 ubiquitin ligase complexes, BTB proteins play pivotal roles in fine-tuning these cold responses through targeted ubiquitination, as well as transcriptional and epigenetic regulation [96].

In *Arabidopsis thaliana*, BTB proteins are integral to the perception of prolonged low-temperature signals, such as those experienced during vernalization, and their integration with plant developmental transition—particularly flowering. Specifically, the BTB proteins LRB1 (LIGHT-RESPONSE BTB1) and LRB2 form a CRL3 complex with CUL3A to mediate the degradation of FRIGIDA (FRI), a key activator of the floral repressor FLOWERING LOCUS C (FLC). Clearance of FRI during vernalization is essential for the epigenetic silencing of FLC, thereby enabling plants to flower in spring after overwintering. Intriguingly, the cold-induced transcription factor WRKY34 upregulates CUL3A expression, further accelerating FRI degradation and promoting timely flowering. This mechanism exemplifies how BTB proteins transduce persistent environmental cues (prolonged cold) into stable epigenetic memory and developmental reprogramming [97].

Beyond modulating prolonged cold responses, the predominant function of BTB proteins in acute cold stress is to act as negative regulators that prevent the excessive or untimely activation of the C-repeat Binding Factor (CBF) pathway. This regulatory logic is highly conserved across various crop species. In tomato (*Solanum lycopersicum*), cold stress induces the expression of the SlBTB19, which specifically interacts with the transcription factor SlWRKY2 and targets it for ubiquitin-mediated degradation. Since SlWRKY2 directly binds to the promoters of CBF1 and CBF3 to activate their transcription, its degradation results in suppression of the CBF signaling cascade. Consequently, SlBTB19-overexpressing plants exhibit hypersensitivity to cold, whereas *slbtb19* knockout mutants display enhanced cold tolerance, establishing SlBTB19 as a molecular “brake” on the CBF pathway [98].

Similarly, in apple (*Malus domestica*), the MdBT2 protein negatively modulates cold tolerance by recognizing and promoting the degradation of the R2R3-MYB transcription factor MdMYB23. This, in turn, indirectly represses the expression of downstream MdCBF1 and MdCBF2 genes. Notably, MdMYB23 also regulates proanthocyanidin biosynthesis, suggesting that MdBT2 coordinates both stress adaptation and secondary metabolism under cold conditions [72].

Additionally, transcriptomic studies in economically important crops such as pepper (*Capsicum annuum*), cucumber (*Cucumis sativus*), and potato (*Solanum tuberosum*) have shown that numerous BTB genes are significantly upregulated under cold stress, implying conserved roles in cold resistance networks. For instance, CaBPM4 in pepper has been implicated in enhancing cold tolerance, potentially by boosting peroxidase (POD) activity and reducing malondialdehyde (MDA) accumulation [99,100,101].

**Figure 3:**
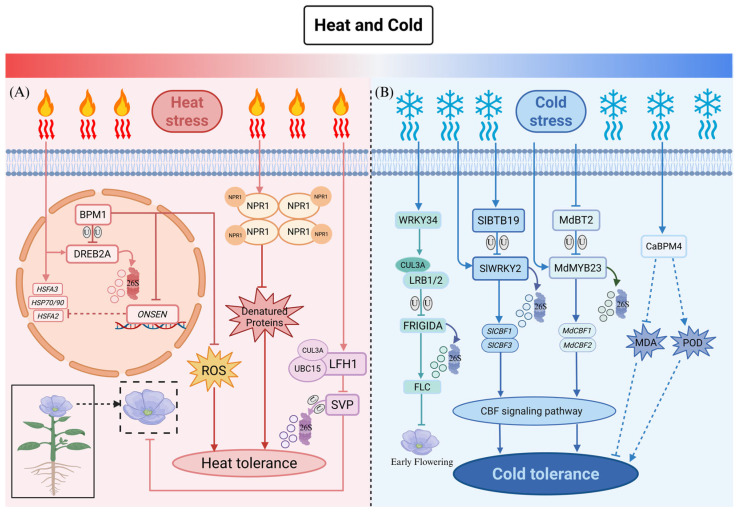
BTB proteins function as central regulators in plant responses to temperature stress. (**A**) Heat Stress: In *Arabidopsis*, BPM1 targets the transcriptional activator DREB2A for degradation to prevent growth retardation associated with its constitutive accumulation. Under heat stress, this degradation persists despite the transcriptional induction of DREB2A, establishing a dynamic equilibrium. Although heat-responsive genes (HSFA3, HSP70/90) are strongly induced after heat treatment, BPM1 overexpression suppresses the induction of HSFA2 and ONSEN. LFH1 promotes early flowering by degrading the repressor SVP. Additionally, NPR1 oligomers act as molecular chaperones to prevent protein aggregation. (**B**) Cold Stress: (1) Flowering: Cold-induced WRKY34 activates the CRL3-LRB1/2 complex to degrade FRIGIDA (FRI), leading to the silencing of FLC to promote vernalization. (2) CBF Pathway: SlBTB19 (tomato) and MdBT2 (apple) negatively regulate cold tolerance by degrading the positive regulators SlWRKY2 and MdMYB23, respectively, thereby attenuating downstream CBF expression. (3) Physiology: CaBPM4 (pepper) enhances cold tolerance by reducing MDA levels and increasing POD activity. Solid lines indicate direct regulation; dashed lines represent indirect associations or physiological outcomes; T-bars indicate inhibition; U denotes ubiquitination. This schematic represents a proposed working model synthesized from established literature. This figure was drawn using Microsoft PowerPoint.

Just as BTB proteins elegantly fine-tune the balance between cold stress resilience and developmental progression (Figure 3), they employ an equally sophisticated regulatory logic to perceive and counteract severe ionic and osmotic imbalances.

#### 3.1.4. Role of BTB Proteins in Plant Salt Stress

Driven by excessive sodium (Na^+^) and chloride (Cl^−^) accumulation, soil salinization imposes a dual challenge of osmotic and ionic stress that disrupts the homeostasis of essential ions like calcium (Ca^2+^) and potassium (K^+^) [102,103]. Moreover, this severe ionic imbalance triggers ROS-induced programmed cell death [104,105]. As a result, excessive salinity severely inhibits critical developmental processes across major crops [106,107,108,109]. To mitigate these detrimental impacts, plants deploy multifaceted adaptive strategies, including the regulation of Na^+^/K^+^ balance, antioxidant activation, and robust salt-responsive signaling cascades [110].

Within this complex response network, BTB proteins, acting as substrate-recognition subunits of the Cullin3 (CUL3)-based E3 ubiquitin ligase (CRL3) complex, serve as central executors and modulators of salt tolerance. Their roles span both positive activation and negative feedback, enabling precise control over the amplitude, duration, and specificity of salt stress responses. A subset of BTB proteins functions as positive regulators that directly activate defense signaling under saline conditions. A paradigmatic example is AtSIBP1 (*Arabidopsis thaliana* stress-induced BTB protein 1), whose expression is strongly induced by both salt stress and abscisic acid (ABA). Loss-of-function sibp1 mutants exhibit hypersensitivity to salt, marked by excessive ROS accumulation and reduced survival, whereas AtSIBP1-overexpression confers enhanced salt tolerance. Mechanistically, AtSIBP1 orchestrates a broad transcriptional reprogramming that upregulates genes involved in osmoprotection (RD29A), membrane stabilization (COR15A), and ROS scavenging (APX2) [23].

Similarly, in rice, the OsBTBZ1 gene is preferentially upregulated in salt-tolerant cultivars under saline conditions. Heterologous expression of OsBTBZ1 in *Arabidopsis* significantly enhances the tolerance to both salt and ABA, while mutation of its *Arabidopsis* homolog, Atbt3, results in salt hypersensitivity. These findings suggest that OsBTBZ1 likely promotes salt tolerance through an ABA-dependent signaling pathway, reinforcing the conserved role of BTB proteins as positive effectors in stress adaptation [111].

In contrast, another class of BTB proteins acts as “molecular brakes”, tempering stress responses to avoid the fitness costs of chronic defense activation. In rice, this regulatory logic is exemplified by the OsWRKY42–OsCUL1–OsMBTB32 module. Under salt stress, OsMBTB32 suppresses root and shoot growth. However, the transcription factor OsWRKY42 is simultaneously activated and induces OsCUL1 expression. The resulting OsCUL1 protein then interacts with OsMBTB32, effectively neutralizing its inhibitory function. This multi-layered cascade enables fine-tuned modulation of growth-defense trade-offs during salt stress [28].

Strategically exploring this degradation-based regulation offers promising avenues for crop improvement. BPM proteins, a well-characterized MATH-BTB subfamily, recognize specific degron motifs (e.g., PEST and SPOP motifs) in stress-related transcription factors (e.g., HB, MYB, MYC, AP2/ERF), and target them for CRL3-mediated proteolysis. Remarkably, transient expression of a synthetic PEST motif-containing peptide in rapeseed (*Brassica napus* cv. Westar) competitively saturated BPM substrate-binding sites, thereby stabilizing endogenous transcription factors normally degraded by BPM. This intervention not only enhanced salt tolerance but also increased oil yield, demonstrating the biotechnological potential of modulating BTB-mediated proteostasis for simultaneous stress resilience and agronomic performance [112].

Further underscoring the functional versatility of the BTB family, NPR1 (Nonexpressor of Pathogenesis-Related Genes 1) exemplifies a protein that integrates biotic and abiotic stress responses through spatiotemporally dynamic roles. Containing the BTB-ANK domain, NPR1 homologs are conserved from *Arabidopsis* to maize (*Zea mays*) and function as pivotal signaling hubs [34]. During early salt stress, NPR1 is synthesized de novo and translocates into the chloroplast stroma, where it acts as a molecular chaperone and redox sensor, safeguarding the proteostasis of photosynthesis machinery. As ROS levels rise, the oxidative environment triggers NPR’s conformational shift: it depolymerizes into monomers and translocates to the nucleus. There, it resumes its canonical role as a transcriptional co-activator, partnering with TGA transcription factors to induce expression of salt tolerance genes, such as TGA1/2, PR1/2/5, RbcS, CABs and SOD. By shuttling between organelles in response to cellular redox status, NPR1 directly couples physiological state to transcriptional output, achieving seamless integration of protection and regulation [113,114].

Genome-wide analyses further support the broad involvement of BTB proteins in salt adaptation. In potato (*Solanum tuberosum*), 34 StBTB genes have been identified, with multiple members implicated in salt stress responses. These findings provide a valuable genetic foundation for the molecular breeding of salt-tolerant potato varieties [101].

**Figure 4:**
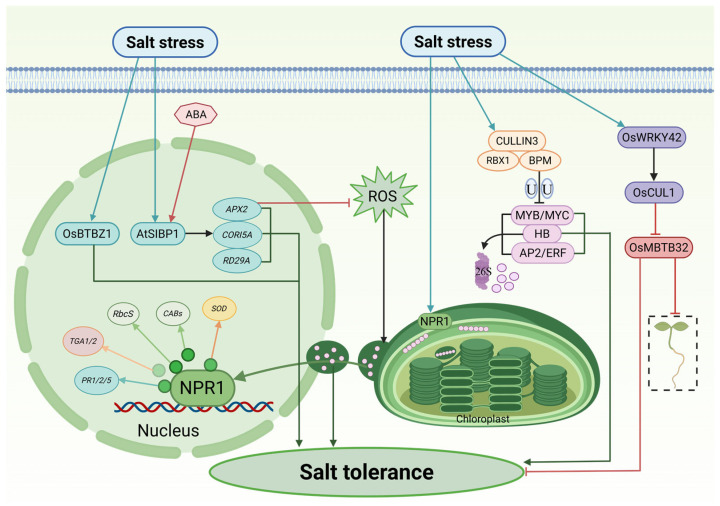
BTB proteins orchestrate the plant response to salt stress through diverse regulatory modules. Positive regulation by OsBTBZ1 and AtSIBP1: Under salt stress, OsBTBZ1 is upregulated to promote salt tolerance. Similarly, AtSIBP1, induced by salt and ABA, activates the expression of stress-responsive genes (e.g., RD29A, COR15A) and the antioxidant enzyme gene APX2. The latter helps scavenge reactive oxygen species (ROS), thereby alleviating oxidative damage. Spatiotemporal regulation by NPR1: NPR1 acts as a dual-regulator responding to ROS accumulation. Upon stress perception, NPR1 translocates to the chloroplast to maintain protein homeostasis. It also moves into the nucleus to function as a transcriptional co-activator, driving the expression of defense-related genes (TGA1/2, PR1/2/5), photosynthesis-related genes (RbcS, CABs), and the antioxidant gene SOD. Negative regulation and fine-tuning: (1) The OsWRKY42–OsCUL1–OsMBTB32 module in rice: The transcription factor OsWRKY42 activates OsCUL1 expression; the resulting ubiquitin ligase component subsequently degrades the repressor protein OsMBTB32. Since OsMBTB32 negatively regulates plant growth and tolerance, its degradation releases this inhibition. (2) The BPM–CUL3 complex: BPM proteins function as substrate adaptors that mediate the ubiquitin-dependent degradation of key transcription factors (MYB/MYC, HB, AP2/ERF), thereby fine-tuning the salt stress response. Arrows indicate promotion or activation; red lines with flat heads indicate inhibition; U denotes ubiquitination. This schematic represents a proposed working model synthesized from established literature. This figure was drawn using Microsoft PowerPoint.

Although the aforementioned mechanisms highlight the indispensability of BTB proteins in mitigating diverse abiotic constraints such as soil salinization (Figure 4), their regulatory versatility extends equally to the perception and transduction of vital environmental cues like light.

### 3.2. Role of BTB Proteins in Plant Light Signal Transduction

Light represents the most critical environmental cue governing the plant life cycle, enabling plants to sense and respond to dynamic changes in their surroundings and to precisely regulate growth and development [115]. Beyond serving as the energy source for photosynthesis, light acts as an informational signal that orchestrates a wide array of processes—including photomorphogenesis, photoperiodic responses, flowering time, and adaptation to variations in light quality (e.g., red, blue, and UV-B) and intensity [116,117]. These responses are mediated by a sophisticated network of photoreceptors that transduce specific light signals into developmental and physiological outputs. For instance, the molecular basis of photoperiod-regulated flowering is well established, with the CONSTANS (CO)–FLOWERING LOCUS T (FT) module serving as a central regulatory hub that promotes floral transition under inductive day lengths [118].

As core substrate-recognition subunits of Cullin3 (CUL3)-based E3 ubiquitin ligase (CRL3) complexes, BTB proteins play multilayered and pivotal roles in plant light signal transduction. By selectively mediating the ubiquitination and degradation of key components within light signaling pathways, they ensure the precision, timing, and amplitude of plant responses to light cues [18,45,119,120].

At the apex of the light signaling hierarchy, BTB proteins directly modulate the activity and stability of the photoreceptors themselves, constituting a fundamental regulatory mode. In blue light signaling, members of the BTB-NPH3/RPT2-like (NRL) family—such as NPH3 (NONPHOTOTROPIC HYPOCOTYL 3) and RPT2 (ROOT PHOTOTROPISM 2)—function as direct interactors of the blue light receptors phototropins (Phot1/Phot2). They regulate diverse physiological outputs, including phototropism, stomatal opening, leaf flattening, chloroplast movement, and auxin transport patterns [14,15,119,121]. Notably, this regulation operates as a light intensity-dependent “dual-mode switch”. Under low-intensity blue light, the CRL3NPH3 complex mediates the mono- or multi-ubiquitination of Phot1. Rather than triggering degradation, this modification acts as an activation signal that promotes clathrin-mediated endocytosis of Phot1 from the plasma membrane to the cytoplasm, thereby initiating downstream phototropic signaling. In contrast, under high-intensity blue light, the complex induces poly-ubiquitination, targeting PHOT1 for 26S proteasome-mediated degradation. This serves as a negative feedback mechanism that attenuates signaling under excessive irradiance, effectively “desensitizing” or “resetting” the system to prevent overactivation [119].

Beyond blue light perception, BTB proteins also critically regulate red/far-red light signaling. The LIGHT-RESPONSE BTB1/2 (LRB1/2) proteins act as substrate adapters for the CRL3LRB complex and specifically recognize phytochrome B (phyB) and phyD, targeting them for ubiquitin-dependent degradation. Through this mechanism, LRB proteins negatively regulate virtually all phyB-mediated processes, including seed germination, shade avoidance, and flowering time. Recent studies have further elucidated a critical role for LRB-mediated phyB degradation in maintaining signaling homeostasis: under continuous red light, the absence of LRB activity leads to the overaccumulation of phyB, which in turn induces intron retention in PIF3 (PHYTOCHROME-INTERACTING FACTOR 3) mRNA, thereby inhibiting PIF3 protein translation. Consequently, LRB proteins not only directly limit photoreceptor abundance but also indirectly safeguard the translational homeostasis of key transcription factors within the light signaling pathway by preventing the overactivation of phyB [16,17,18].

Moreover, emerging evidence indicates that LRBs specifically recognize and interact with photoexcited and phosphorylated CRY2 to mediate its ubiquitin-dependent degradation. Through this mechanism, LRBs negatively regulate CRY2 protein abundance to prevent excessive blue light signaling, thereby modulating photomorphogenesis. This underscores their role as integrative nodes that bridge distinct photoreceptor pathways [122].

In addition to regulating photoreceptors, BTB proteins exert multi-tiered fine-tuning of light responses by governing the stability of downstream signaling components and transcription factors. A compelling example comes from apple (*Malus domestica*), where MdBT2 acts as a “molecular switch” for light signaling. Under low light conditions, highly expressed MdBT2 functions as a substrate adapter for the E3 ubiquitin ligase complex to mediate the ubiquitin-dependent degradation of the positive transcription factor MdTCP46. This process attenuates MdTCP46-mediated activation of the core transcription factor MdMYB1, thereby repressing the expression of anthocyanin biosynthetic genes. Conversely, high light signaling significantly suppresses MdBT2 transcription and promotes its protein degradation. This relieves the repression on MdTCP46, allowing it to accumulate and synergize with MdMYB1 to efficiently activate anthocyanin biosynthesis, conferring photoprotection under high irradiance [123].

Furthermore, MdBT2 physically interacts with another central E3 ligase, COP1 (CONSTITUTIVELY PHOTOMORPHOGENIC 1), and stabilizes it by inhibiting its auto-ubiquitination. In *Arabidopsis*, COP1 promotes the degradation of key positive regulators of photomorphogenesis and anthocyanin accumulation, such as HY5 and PAP1/2 [124,125]. In apple, stabilized COP1 more efficiently targets MdMYB1 for degradation, thereby repressing anthocyanin synthesis. Given the strong link between anthocyanin production and photoperiodic cues, this regulatory axis provides a mechanistic foundation for light-controlled pigment accumulation. Within this network, MdBT2 functions not only as a passive adapter but as an active “regulator of regulators”, exemplifying the layered complexity of ubiquitin-mediated control in light signaling [126].

Importantly, BTB proteins are themselves direct targets of light regulation, forming dynamic feedback loops that enhance signaling plasticity [14,18,45,119]. The promoters of numerous BTB genes are enriched in light-responsive elements (LREs), such as the G-box motif, enabling their transcription to be rapidly and reversibly modulated by changes in light quality and intensity. This allows plants to dynamically adjust the intracellular abundance of BTB proteins in accordance with ambient light conditions, thereby more precisely calibrating the overall sensitivity and output of the light signaling network [15,16,17,18,19,20,21,22,23,24,25,26,27,28,29,30,31,32,33,34,35,36,37,38,39,40,41,42,43,44,45,46,47,48,49,50,51,52,53,54,55,56,57,58,59,60,61,62,63,64,65,66,67,68,69,70,71,72,73,74,75,76,77,78,79,80,81,82,83,84,85,86,87,88,89,90,91,92,93,94,95,96,97,98,99,100,101,102,103,104,105,106,107,108,109,110,111,112,113,114,115,116,117,118,119,120,121,122,123,124,125,126,127].

**Figure 5:**
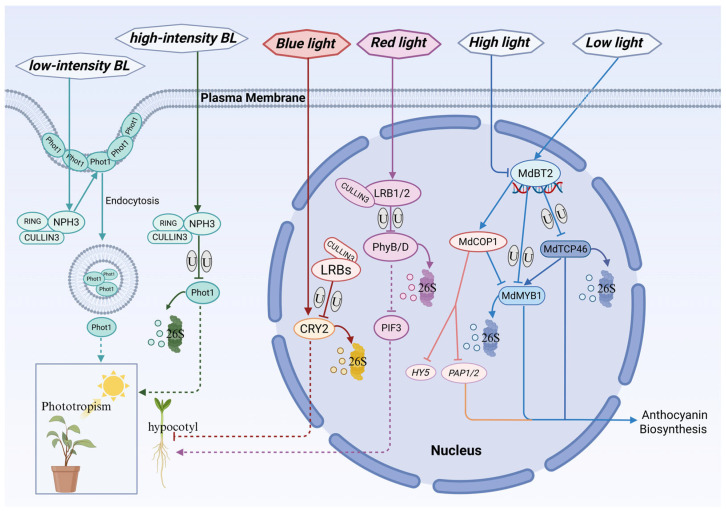
Multilayered regulatory network of BTB proteins in plant light signal transduction. The CRL3-NPH3 complex acts as a dual-mode switch for the blue light receptor Phot1. Under low-intensity blue light (BL), NPH3 mediates the mono/multi-ubiquitination of Phot1, promoting its endocytosis and subsequent phototropic growth. Conversely, under high-intensity BL, NPH3 induces Phot1 poly-ubiquitination and degradation via the 26S proteasome to desensitize the signal. LRB1/2 proteins function as feedback attenuators. They form CRL3 complexes to mediate the ubiquitin-dependent degradation of red light receptors (PhyB/D) and the blue light receptor (CRY2), often in coordination with the signaling hub PIF3, thereby regulating photomorphogenesis. MdBT2 negatively regulates anthocyanin biosynthesis under varying light conditions through two distinct mechanisms: (1) degrading the positive transcription factor MdTCP46, which reduces MdMYB1 expression; and (2) stabilizing the E3 ligase MdCOP1 by inhibiting its auto-ubiquitination. Stabilized MdCOP1 subsequently targets the key activator MdMYB1 (and likely HY5/PAP1/2) for degradation, repressing anthocyanin accumulation. Solid lines indicate direct regulation or conversion; dotted lines represent biological responses or indirect associations; U denotes ubiquitination. This schematic represents a proposed working model synthesized from established literature. This figure was drawn using Microsoft PowerPoint.

Beyond empowering plants with the flexibility to mount appropriate physiological responses to fluctuating light environments (Figure 5), this multi-layered regulatory capacity enables BTB proteins to act as core decision-makers throughout the entire plant developmental continuum.

### 3.3. Role of BTB Proteins in Plant Development

The plant life cycle represents a continuous and precisely orchestrated continuum spanning from vegetative growth to reproductive development, governed by an intricate interplay of hormonal signals, photoperiodic cues, and environmental stimuli. As core substrate adapters for Cullin-RING Ligase 3 (CRL3) complexes, BTB proteins exert pivotal regulatory functions throughout the entire plant lifespan by modulating the stability, activity, and signaling dynamics of their target proteins. Their influence spans every critical developmental stage, from seed germination and the establishment of root system architecture to the floral transition, and ultimately to fruit ripening and quality formation.

At key developmental checkpoints—particularly seed germination and flowering time—BTB proteins act as molecular “decision-makers”, ensuring these transitions occur only under optimal internal and environmental conditions. To balance the trade-off between dormancy and germination, *Arabidopsis* BTB proteins AHT1 (ABA-HYPERSENSITIVE BTB/POZ PROTEIN 1) and BTB-A2s form a sophisticated regulatory network within the ABA signaling pathway. AHT1 expression is induced by ABA and depends on transcriptional activation by core bZIP transcription factors such as ABI5 and AREB1. Paradoxically, AHT1 functions in a negative feedback loop by promoting the ubiquitin-dependent degradation of ABI5, thereby limiting ABI5 accumulation and suppressing the expression of downstream germination-inhibitory genes (e.g., RD29B). This mechanism prevents excessive ABA-mediated inhibition of germination [128]. In parallel, the *Arabidopsis* BTB-A2s proteins (BTB-A2.1/2.2/2.3) negatively regulate the ABA signaling pathway by targeting the kinase SnRK2.3 for degradation, further attenuating the inhibitory effect of ABA on seed germination [25]. Collectively, these mechanisms ensure that seeds break dormancy and germinate only under favorable conditions, striking a delicate balance between stress resilience and developmental progression. In regulating the floral transition, BTB proteins integrate multiple environmental signals to synchronize flowering with seasonal changes. On one hand, BPM proteins promote flowering by mediating the degradation of floral repressors such as MYB56, thereby relieving repression of the florigen gene FLOWERING LOCUS T (FT) and facilitating floral initiation [19]. On the other hand, during vernalization, the transcription factor WRKY34 binds to the W-box element in the CUL3A promoter to upregulate CUL3A expression. Consequently, the assembled CRL3 complex (involving LRB1/2) targets the central floral regulator FRIGIDA (FRI) for degradation. FRI clearance reduces expression of FLOWERING LOCUS C (FLC), thereby fine-tuning flowering time in response to prolonged cold [97].

In shaping plant architecture, BTB proteins precisely sculpt both below-ground and above-ground structures by modulating hormonal signaling pathways and cell cycle progression. At a fundamental level, the *Arabidopsis thaliana* protein ABAP1 (Armadillo BTB *Arabidopsis* Protein 1) unveils how BTB proteins couple cell cycle control with organogenesis. ABAP1 negatively regulates DNA replication via a dual mechanism: First, by physically interacting with components of the pre-replication complex (pre-RC) (e.g., AtORC1a and AtCDT1a/b), it impedes the pre-RC assembly on chromatin, thereby inhibiting mitotic DNA replication. Second, by forming a complex with the transcription factor AtTCP24, it binds to the promoters of AtCDT1a/b to repress their transcription. This dual regulation directly links cell cycle progression with leaf morphogenesis, ensuring proper organ development [129].

Building upon these fundamental developmental controls, BTB proteins also serve as primary negative regulators of root system architecture (RSA) in agricultural crops. In chrysanthemum (*Chrysanthemum morifolium*), CmBT1 inhibits primary root growth by mediating the degradation of the MADS-box transcription factor CmANR1. Concurrently, this degradation downregulates CmPIN2, a direct transcriptional target of CmANR1, collectively suppressing root development [17]. Furthermore, multiple CmBT family members (e.g., CmBT1 and CmBT5) broadly inhibit the formation of adventitious roots (ARs) and root hairs by modulating nitrate assimilation, amino acid metabolism, and the auxin and jasmonic acid (JA) signaling pathways [130].

In apple (*Malus domestica*), MdBT2 orchestrates AR development through a dual regulatory mechanism. First, it directly targets the auxin response factor MdARF8 for ubiquitin-mediated degradation, abolishing MdARF8’s ability to activate downstream genes such as MdGH3.1/3.2/3.5/3.6. Simultaneously, MdBT2 stabilizes the auxin repressor MdIAA3, enhancing its heterodimerization with MdARF8 and other MdARFs (e.g., MdARF5/6/7/19), which further represses the expression of AR-related genes. Second, MdBT2 acts as a molecular scaffold, strengthening the interaction between MdIAA3 and MdARF8 and facilitating the assembly of a ternary “MdBT2–MdIAA3–MdARF8” complex. This elegantly integrates the ubiquitin-proteasome system with the canonical TIR1/AFB–AUX/IAA auxin signaling module, synergistically inhibiting adventitious rooting. Collectively, these findings highlight BTB proteins as critical regulators of root development and promising molecular targets for crop root system improvement [131].

In crops, BTB proteins serve as pivotal determinants of key agronomic traits related to fruit yield and quality. In apple, MdBT2 functions as a “dual-faced modulator” of fruit quality. On one hand, it degrades the transcription factor MdMYB73, attenuating its activation of malate accumulation genes (e.g., MdALMT9, MdVHA-A, MdVHP1), thereby reducing fruit acidity [132]. On the other hand, MdBT2 dynamically regulates pigment biosynthesis in response to light intensity through the MdBT2–TCP46–MYB1 module. Under high light, MdBT2 expression is suppressed, leading to stabilization of MdTCP46. Accumulated MdTCP46 enhances the DNA-binding activity of MdMYB1 to promoters of anthocyanin biosynthetic genes (e.g., MdDFR and MdUF3GT), activating the pathway and promoting fruit coloration [123]. Beyond quality traits, BTB proteins also govern fruit defense and postharvest performance. For instance, MdPOB1 in apple targets the disease resistance activator MdPUB29 for degradation, suppressing hydrogen peroxide (H_2_O_2_) accumulation and the salicylic acid (SA) signaling pathway, leading to the downregulation of key SA biosynthetic genes (MdEDS1, MdPAD4, MdPAL) and signaling genes (MdNPR1, MdPR1, MdPR5), thereby negatively regulating fruit disease resistance. This illustrates how BTB-mediated proteolysis can influence both biotic stress responses and fruit physiology [133].

In tomato (*Solanum lycopersicum*), SlBTA2 positively regulates cuticle biosynthesis through a coordinated transcriptional program. Mechanistically, it mediates the ubiquitin-dependent degradation of the transcriptional repressor SlMYB41, thereby relieving the repression of genes involved in cutin biosynthesis (SlLACS2, SlANL2b, SlCYP86A68), wax biosynthesis (SlKCS1, SlCER1-2), and lipid transport (SlABCG11). By orchestrating these pathways, SlBTA2 enhances peel integrity and reduces postharvest water loss, significantly influencing fruit development and shelf life, and offering a promising avenue for future fruit quality improvement [134].

More broadly, BTB proteins function as versatile signaling platforms that integrate diverse endogenous and exogenous cues to coordinate plant growth and development. For instance, ETO1 interacts with CUL3 to form a functional CRL3 complex that regulates ethylene biosynthesis during embryogenesis by targeting ACS5, the key rate-limiting enzyme, for degradation [135]. In *Arabidopsis*, AtLRB3 (Light-Response BTB protein 3) is implicated in sensing nitric oxide (NO) signals; by modulating the stability of photomorphogenesis regulators (such as phytochromes or other signaling proteins), it potentially integrates photoperiod and NO perception [136]. In apple, MdBT2 further exemplifies this integrative capacity by coupling gibberellin (GA) signaling with nitrogen nutrient status through the degradation of the DELLA protein MdRGL3a, thereby synergistically promoting plant growth [137].

**Figure 6:**
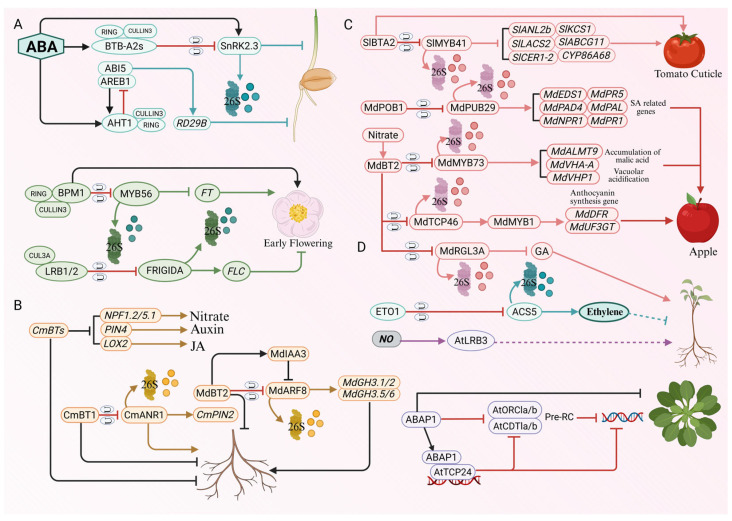
Regulatory networks of BTB proteins orchestrating plant growth and development. (**A**) Seed germination and flowering time: ABA signaling induces AHT1, which negatively feeds back by degrading ABI5, while BTB-A2s degrade SnRK2.3 to fine-tune germination. For flowering, BPM1 degrades the repressor MYB56, relieving the repression of FT; The CRL3-LRB1/2 complex degrades FRIGIDA (FRI), thereby reducing FLC expression. (**B**) Shaping plant architecture and organogenesis: In chrysanthemum, CmBT1 inhibit root growth by degrading CmANR1, thereby downregulating CmPIN2. In apple, MdBT2 inhibits adventitious rooting by degrading MdARF8 and stabilizing MdIAA3. In *Arabidopsis*, ABAP1 negatively regulates DNA replication and leaf development by blocking pre-RC assembly (AtORC1a, AtCDT1a/b) via interaction with AtTCP24. (**C**) Fruit yield, quality, and defense: In apple, MdBT2 functions as a dual-modulator: it degrades MdMYB73 to reduce fruit acidity, and degrades MdTCP46 to regulate anthocyanin synthesis via MdMYB1. MdPOB1 negatively regulates disease resistance by degrading MdPUB29, which suppresses SA signaling and ROS accumulation. In tomato, SlBTA2 promotes cuticle formation by degrading the repressor SlMYB41, thereby relieving the repression of cutin and wax biosynthesis genes. (**D**) Integration of diverse signaling pathways: ETO1 targets ACS5 to control ethylene biosynthesis. AtLRB3 is involved in NO signaling by modulating photomorphogenesis regulators. MdBT2 integrates GA and nitrogen signaling by degrading the DELLA protein MdRGL3a to promote plant growth. Solid lines indicate positive regulation/activation; T-bars indicate negative regulation/inhibition; “U” and “26S” denote ubiquitin-proteasome system mediated degradation. This schematic represents a proposed working model synthesized from established literature. This figure was drawn using Microsoft PowerPoint.

By serving as molecular hubs that bridge genotype with phenotype across virtually every developmental node (Figure 6), BTB proteins enable dynamic adaptation to both internal cues and external fluctuations, establishing the profound functional paradigms synthesized in the following concluding remarks.

## 4. Concluding Remarks and Future Perspectives

By synthesizing the diverse mechanisms discussed across previous sections, a unified regulatory logic emerges: BTB proteins act as dynamic molecular hubs that seamlessly bridge environmental perception with physiological adaptation and developmental progression. As the pivotal specificity-determining subunits of Cullin-RING Ligase 3 (CRL3) complexes, their biological function extends far beyond acting as static structural bridges. Instead, they dynamically dictate plant phenotypic plasticity. Specifically, the previously fragmented insights from abiotic stress resilience, light signaling, and plant architecture converge into four overarching functional paradigms:

As “Molecular Brakes” and “Accelerators”: By mediating the degradation of either negative or positive regulators within signaling pathways (e.g., CBF activators, SnRK2 kinases in ABA signaling), they precisely initiate, terminate, or calibrate response intensities, ensuring an optimal trade-off between plant growth and defense.

As Modulators of Signal Perception: They directly regulate photoreceptor stability (e.g., phyB, phot1) through mechanisms such as “dual-mode switches” or feedback-driven degradation, dynamically tuning plant sensitivity to the light environment.

As Executors of Developmental Switches: By degrading key regulators (e.g., FRI, ANR1, ABI5), they enforce irreversible transitions at critical life cycle checkpoints (e.g., vernalization, seed germination).

As Integrators of Signaling Pathways: They function as core nexus points that couple distinct signaling pathways (e.g., GA and nitrogen, hormones and stress signals), bridging endogenous developmental programs with external environmental inputs.

Despite substantial advances, several critical questions remain unexplored. First, the molecular basis of substrate recognition specificity among BTB proteins is incompletely understood. In particular, how auxiliary domains (e.g., BACK, TAZ), contribute to target selectivity and binding affinity warrants deeper mechanistic investigation. Second, the expansive nature of the BTB protein family often leads to functional redundancy and complementarity, complicating genetic dissection. Future studies should leverage advanced gene editing technologies (e.g., CRISPR/Cas9) to generate higher-order mutants, enabling the systematic unraveling of their interaction networks and regulatory hierarchies. Third, current research remains heavily focused on model species (*Arabidopsis*) and a limited set of horticultural crops. Systematic functional characterization of BTB proteins in other economically important fruit and vegetable crops (e.g., grape, citrus, banana, and legumes) is urgently needed. A multidimensional research framework should be established, integrating species-specific developmental traits and agronomic priorities to maximize translational impact. Furthermore, successful regulatory modules identified in model systems—such as those governing drought resistance, salt tolerance, and flowering time—should be rapidly translated to major staple crops (e.g., rice, maize, wheat, and soybean) to validate and optimize their breeding efficacy across diverse genetic backgrounds. Finally, historical breeding efforts have often encountered growth-defense trade-offs, where enhanced stress resistance compromises yield. However, targeted engineering of specific BTB proteins that govern the stability of key transcription factors now offers a promising route to uncouple this antagonism. This strategy holds great potential for developing “smart crops” that combine robust stress resilience with uncompromised productivity [138].

In conclusion, by structurally acting as specific substrate adaptors within CRL3 complexes, BTB proteins function as pivotal regulatory nodes that govern plant environmental adaptability, growth, and development. Furthermore, they serve as strategic molecular hubs linking environmental perception to crop yield formation. Research on BTB proteins has progressively expanded from model systems to major agricultural crops, underscoring their robust regulatory capacity and immense potential for precision breeding. Future endeavors should focus on the fine-scale dissection of their molecular mechanisms and actively facilitate the translation of basic discoveries into field applications. Such efforts will be instrumental in advancing sustainable agriculture and safeguarding global food security.

## Figures and Tables

**Table 1:** Classification and functions of diverse auxiliary domains in plant BTB proteins.

Auxiliary Domain	Representative Proteins (Species)	Main Biological Functions/Mechanisms	References
ANK	NPR1 (*Arabidopsis thaliana*)	Mediates protein–protein interactions with TGA and TCP transcription factors to regulate systemic acquired resistance (SAR).	[35,36]
TAZ	BT1, BT2 (*Arabidopsis thaliana*)	Recruits histone acetyltransferases (HATs) to remodel chromatin; regulates light-responsive gene expression.	[37,38,39]
MATH	BPMs (*Arabidopsis thaliana*)	Acts as substrate receptors targeting transcription *factors (e.g., ABI5)* for degradation, modulating ABA signaling.	[40,41]
NPH3	NPH3 (*Arabidopsis thaliana s*)	Interacts with phototropin 1 (PHOT1) to regulate phototropism and polar auxin transport	[14]
TPR	BTB-TPR family	Modulates ethylene biosynthesis, gibberellin/cytokinin responses, and ABA/osmotic stress signaling.	[42]
DUF	OsSIDP366/361 (*Oryza sativa*)	Positively regulates salt and drought tolerance via mechanisms involving domains of unknown function (DUF).	[43]
Skp1-like	SCF complex members	Forms part of the SCF (Skp1-Cullin-F-box) E3 ligase complex regulating hormone signaling, circadian rhythms, and floral development.	[44,45]

## Data Availability

The original contributions presented in this study are included in the article. Further inquiries can be directed to the corresponding authors.

## Abbreviations

The following abbreviations are used in this manuscript:

ABA	Abscisic acid
ABAP1	Armadillo BTB Arabidopsis Protein 1
AHT1	ABA-HYPERSENSITIVE BTB/POZ PROTEIN 1
ANK	Ankyrin
APX	Ascorbate peroxidase
AR	Adventitious root
ARF	Auxin response factor
AtSIBP1	Arabidopsis thaliana stress-induced BTB protein 1
bHLH	Basic helix-loop-helix
bZIP	Basic region leucine zipper
BPM	BTB/POZ-MATH
BR	Brassinosteroid
BTB	Broad-Complex, Tramtrack, and Bric-à-brac
CAB	Chlorophyll a/b binding protein
CAT	Catalase
CBF	C-repeat Binding Factor
CO	CONSTANS
COP1	CONSTITUTIVELY PHOTOMORPHOGENIC 1
COR	Cold-regulated
CRL3	Cullin-RING Ligase 3
CRY2	Cryptochrome 2
CUL3	Cullin 3
DREB2A	Dehydration-Responsive Element-Binding Protein 2A
DUF	Domain of Unknown Function
ERF	Ethylene response factor
FLC	FLOWERING LOCUS C
FRI	FRIGIDA
FT	FLOWERING LOCUS T
GA	Gibberellin
GPX	Glutathione peroxidase
HAT	Histone acetyltransferase
HSP	Heat Shock Protein
IAA	Indole-3-acetic acid/Auxin repressor
JA	Jasmonic acid
LFH1	Late Flowering at High temperature 1
LRB	LIGHT-RESPONSE BTB
LRE	Light-responsive element
MATH	Meprin and TRAF Homology
MDA	Malondialdehyde
MYB	Myeloblastosis family transcription factor
NAC	NAM, ATAF1/2, and CUC2
NO	Nitric oxide
NPH3	NONPHOTOTROPIC HYPOCOTYL 3
NPR1	NONEXPRESSOR OF PATHOGENESIS-RELATED GENES 1
NRL	NPH3/RPT2-like
OST1	OPEN STOMATA 1
PCD	Programmed cell death
PHOT1	Phototropin 1
phyB/D	Phytochrome B/D
PIF3	PHYTOCHROME-INTERACTING FACTOR 3
POD	Peroxidase
POZ	Pox virus and Zinc finger
pre-RC	Pre-replication complex
RD29A/B	Responsive to Desiccation 29A/B
ROS	Reactive oxygen species
RPT2	ROOT PHOTOTROPISM 2
RSA	Root system architecture
SA	Salicylic acid
SAR	Systemic acquired resistance
SCF	Skp1-Cullin-F-box
SnRK2.6	SNF1-related protein kinase 2.6
SOD	Superoxide dismutase
SVP	SHORT VEGETATIVE PHASE
TAZ	Transcriptional coactivator with PDZ-binding motif
TCP	Teosinte branched 1/cycloidea/PCF
TGA	TGACG-binding motif
TPR	Tetratricopeptide repeat
UPS	Ubiquitin-proteasome system
